# Sensing and making sense of tourism flows and urban data to foster sustainability awareness: a real-world experience

**DOI:** 10.1186/s40537-021-00442-w

**Published:** 2021-03-24

**Authors:** Catia Prandi, Valentina Nisi, Miguel Ribeiro, Nuno Nunes

**Affiliations:** 1 Department of Computer Science and Engineering, Bologna, Italy; 2 Instituto Superior Técnico, U. of Lisbon, Lisbon, Portugal; 3 ITI/LARSyS, Funchal, Portugal

**Keywords:** Big data, Urban data, Tourism flows, Pervasive infrastructure, Data visualization, Citizens participation

## Abstract

Tourism is one of the world’s largest industries fundamentally arising from mobility as a form of capital. In destination islands that have a delicate ecosystem to maintain, this source of income can become problematic in terms of sustainability. A difficulty in making people aware of this issue is also represented by the fact that such sustainability-related issues (and their causes) are often not “visible” to citizens. To foster awareness about the relationship between sustainability and tourism in well-known destinations, we design a platform that engages users at two levels of participation: i. at the IoT and sensors level, in order to let them becoming providers of big data, deploying and enlarging the pervasive infrastructure; ii. at the (big) data visualization level, with the aim of engaging them in making sense of large volumes of data related to sustainability. This paper presents the design and implementation of a real-world experience where a low-cost collaborative platform made it possible to sense and visualize tourist flows and urban data into a rich interactive map-based visualization, open to the local communities. We deployed our case study in the Madeira archipelago, engaging locals and visitors of the island in two exploratory studies focused on measuring the impact of providing users with meaningful representations of tourism flows and related unperceivable aspects that affect the environmental sustainability. Analysing the findings of the two studies, we discuss the potentiality of using such a system to make sense of big data, fostering awareness about sustainability issues, and we point to future open challenges about citizens’ participation in sensing and making sense of big data.

## Introduction

Tourism is a continuously growing and important sector for many regions and countries worldwide for which it is a central source of welfare. In fact, tourism is one of the world’s largest industries fundamentally arising from mobility as a form of capital [1]. As such, tourism is one of the undisputed key issues of modern life, affecting people’s well-being and quality of life, while, at the same time, impacting significantly the environment and the sustainable development [2–4]. Thanks to digital networked technologies, the environment is becoming increasingly interconnected and vast amounts of data are accessible to produce new insights and knowledge about the world we live in (see, for examples [5–7]). These ubiquitous technologies provide researchers with an opportunity to study the overall impact of complex human and economic activities such as tourism. This research is particularly relevant for islands, not only because they are vulnerable to climate change impacts, but also because they provide unique conditions to test complex variables in a controlled environment. Through the project here described we wanted to better understand the impact on locals and visitors when exposed to meaningful representations of the tourism flows on their own locales: what if people could visualize and make sense of touristic flows (some of which are even unnoticeable)?

To this end, in this paper, we describe the design and deployment of a platform, composed by (i) Beanstalk, a low-cost community-based pervasive collaborative infrastructure to sense the presence and movement of people [8], and (ii) ViTFlow, which combines the data collected by the Beanstalk sensing infrastructure and other urban data sets, with visualization technologies, making tourism flows and some related data (such as, common mobility paths, tourist distribution, energy consumption and CO$_{2}$ emission) openly visible to both locals and visitors [9]. The case study has been deployed in an Atlantic Ocean archipelago, Madeira, one of the outermost regions of Europe. To note is that such an area accounts for 80% of the biodiversity of the European continent [10] and provides a unique testbed for testing these technologies.

ViTFlow, harnesses Beanstalk, arises from studies on understanding objects’ trajectories recorded by GPS enabled devices [11, 12], social media information provided by publicly shared tweets and photos [13, 14], and network-based tracking technologies recorded by wireless networks [15–17]. These systems are all generating large volumes of data, enabling new understandings of human mobility. Conversely, new and cheap Internet of Things (IoT) devices are able to sense and transmit environmental data wirelessly as they weave themselves into the fabric of our everyday lives [18]. They enable an unprecedented analysis of many different parameters affecting the environment and human wellbeing and quality of life [19]. From greenhouse gas emissions, impacting on the increase of air pollution, generating N$_{2}$O and CO$_{2}$ and strongly affecting air quality, to changing environmental conditions such as temperature, humidity, and rainfall, all can be monitored and estimated at spatial-temporal resolutions. The big data flows collected in such ways need to be aggregated and displayed in a fashion that they can deliver value. Information visualization techniques come into play to try to solve this issue and deal with the flood of information, helping casual users in making sense of vast amounts of raw data, fostering participation’ reflection, and supporting thinking [20–22].

Inspired by research in HCI and sustainability [23, 24], pervasive sensing and citizen science [25, 26], and information visualization [20, 27–29], we designed and developed ViTFlow as proof of concept to prove the urgent need to facilitate communities in making sense of a variety of data. Our goal was to stimulate more informed opinions about the environmental consequences and the ecological footprint caused by tourism flows. To achieve this, we exploited Beanstalk, a low-cost infrastructure to collect mobility flows of tourists and locals, environmental conditions and air quality. Then, we augmented such big data with related data (e.g., weather condition), and we presented all the data in a rich map-based public visualization available to all the interested communities, to increase their awareness about sustainable issues related to their surroundings. The contributions of this paper are:

- A real-world case study that exploits a dataset of more than four-year mobility data (more than 480 million data points) collected across a medium-sized European Island where a low-cost sensing IoT infrastructure is spread in more than 80 points of interest (POIs) and 20 public buses. This infrastructure is capable of collecting data about relevant parameters impacting the sustainability of the island, such as citizens and tourists mobility flows and air quality;
- The design and implementation of a rich map-based interactive interface enabling locals and visitors to easily make sense of complex dynamics, visualizing big data, with the aim of enhancing and informing communities about sustainability concerns related to tourism and the environment;
- An empirical assessment through two user studies of the potentiality of ViTFlow to be used as a tool for raising people and community reflections about unperceivable aspects that impact environmental sustainability.

The paper is structured as follows: we first present a comprehensive section on related work concerning: (i) mobility tracking and analysis, with a particular emphasis on tourists flows; (ii) sensing and monitoring of environmental and urban data, and (iii) raising people and community awareness about the environmental impacts of human activities. Then, we present the design of the entire platform from a technical perspective: we start introducing Beanstalk, the pervasive infrastructure exploited to collect mobility and environmental data in the Madeira archipelago [8], and we continue presenting ViTFlow in detail. In particular, we describe the different techniques implemented to visualize the datasets, exploiting a layered structure, that makes it possible to synchronize different animations of historical datasets in the same visualization. We then move into an analysis of our empirical investigation of ViTFlow through two exploratory studies. Our studies explore how public visualizations of sensed data makes people reflect on the everyday environment. Finally, we discuss the findings from the studies in relation to understanding tourism flows and environmental impacts and we present directions for future experiments.

## Related work

In this section, we focus on related projects and studies that address how we can sense: (i) mobility flows, analysing mobile phone data, GPS traces, information gathered by Wi-Fi technologies or derived from social network usage, but also (ii) environmental and urban data exploiting participatory network and citizen science. Finally, we present studies focused on (iii) raising communities’ awareness about unperceivable aspects that impact environmental sustainability, investigating how information visualization has been used to make sense of data.

### Mobility and social network tracking and analysis

A vast body of research has been conducted looking at how mobility tracking and analysis on urban environments could be used to estimate mobility flows and patterns [30]. During the last two decades, several studies exploited mobile phone data usage to track users and understand their movements [31–34]. For instance, the authors of [35], presented a study showing the usage of aggregate mobile phone data, provided by an Italian telecommunication company, to estimate the main directions followed by people (i.e., to understand how the mass of people distribute in space and time), by computing a suitable approximation of the Wasserstein distance between two consecutive density profiles. In [34], the authors report on an analysis of mobile phone data to ascertain subgroups with different purposes of visit and related spatio-temporal patterns. Lately, some authors took advantage of mobile phone data to investigate mobility patterns during the COVID-19 pandemic the world is experiencing [36–38]. Indeed, using mobile phone data is not always a feasible solution since the collected data are, in most cases, owned by private large corporations and telecom providers.

To overcome this issue, several studies focus of passive Wi-Fi sensing technologies, through Wi-Fi probe requests, to collect human mobility information and infer mobility patterns [39–42]. Recently, the authors of [43] presented an attempt to localize crowds with Wi-Fi probes applying location fingerprinting interpolations from the received signal strength (RSSI) values from previously scanned indoor locations. Another lens to examine passive Wi-Fi sensing data to understand crowd behaviors and discover patterns is through machine learning [44, 45]. Interestingly, some studies proved that, even with a data set that contains a large portion of randomized MAC addresses, it is still possible to draw meaningful results regarding crowd behaviors [46, 47]. Through the Wi-Fi probes analysis is also possible to profiling the user [48]. An interesting method adopted in a study from Cunche et al. [49] uses the information broadcast from 8000 Wi-Fi devices to perform what the authors called SSID profiling. This technique involves analyzing the captured information, focusing on the SSIDs (names of the saved networks on the devices) to associate different devices with social connections to locate people that visit the same places, share the same interests or family bonds. Similar to SSID profiling, the user demographics of certain Wi-Fi locations have also been studied. Methods range from passive scanning [48], to active meta-data access from HTTP accesses [50].

During the last decade, the paradigm of social sensing has been investigated also in the context of tourism and mobility [51–53]. In [54], the authors conceptualized the use of a large-scale participatory sensing network fed with location-based social media systems to infer valuable knowledge about the city dynamics and urban social behaviour. The authors of [13] explore techniques for detecting urban mobility patterns and anomalies by analysing trajectories mined from publicly available geo-positioned social media traces (i.e., Twitter) left by the citizens. Twitter has been investigated also in [55] for real-time traffic event detection. Instead, the authors of [56] exploited Flickr geotagged photographs together with high-resolution land cover mapping to identify different cultural services and their association with specific ecosystems and land cover types.

Some research studies focus on the specific analysis of tourist flows, mostly focusing on exploiting GPS traces [11, 12], and social media sensing [57–59].

Inspired by the presented studies, we implemented a pervasive system for tracking mobility flows (considering both tourists and locals) with the aim to addressing different challenges at once: developing a low-cost, non-intrusive and secure (preserving users privacy, as detailed in [60]) infrastructure, able to cover urban areas served by public transportation systems as well as very isolated and rural areas; engaging the local communities to facilitate the infrastructure deployment and maintenance (i.e., community-based infrastructure).

### Sensing and monitoring environmental and urban data

In the last decades, different research projects exploited citizen science efforts and participatory sensing (including crowdsourcing and crowdsensing) to detect and monitor a vast range of attributes of the environment [61]. Such attributes range from physical characteristics (as urban accessibility [62, 63]), to actual measurements (as noise [64, 65], air quality [19, 66, 67], and so on), including also environmental-related dimensions (as biodiversity issues [68, 69]. In some cases, the participants contribute writing reports about a certain observed condition, such as weather observations [70], hydrological monitoring [71] and daily precipitation [72]. Some researchers designed specific hardware to accomplish a particular goal [73, 74]. In [74] the authors described MyPart, a personal, wearable, portable, and accurate particle sensor under $50 capable of distinguishing and counting differently sized particles. The collected information is shown in a compelling real-time visualization using an iOS app. A similar approach is exploited in the Sensing the Air project, part of the European CITI-SENSE project, aiming to develop sensor-based citizen observatories. During the project, 30 air-quality sensors were deployed in citizens’ homes and public places (and relocated periodically) to continuously monitor the air quality of the local environment [67, 75].

Several projects engaged citizens equipped with smart devices, exploiting the build-in sensors and creating a participatory sensing network [76, 77]. An interesting study that investigates the sensing of environmental conditions using common smart devices, focusing on noise and sound monitoring, is presented in [64]. More recently, the authors of [78], analyzed a mobile application to collect geotagged images of mosquitoes. During the COVID-19 pandemic, smartphone sensors and crowdsourcing have been employed to collect respiratory sound data [79], and contact tracing data [80].

The presented projects are interesting examples of how it is possible to engage citizens and creating a participatory sensing network to measure a parameter of interest. In our system, we followed a two-level approach to involve citizens in the project. Firstly, we deployed the infrastructure simple inviting citizens, including people working for public entities and owners of places, to install our sensors station for passive Wi-Fi tracking in the point of interest (such as restaurants, bars, malls, museums, the airport, and so on). Second, we implemented ViTFlow to deliver data back to local communities through simple and meaningful visualizations, to stimulate discussions and reflections about sustainability.

### Raising people and community environmental awareness through data visualization

The data and information visualizations are tremendous tools to convey messages and allow users to become more informed about specific issues [20, 81]. In 2007, Pousman et al. stated the importance of information visualization targeted to casual users (in contrast with expert users), extending the definition of InfoVis with four categories, such as ambient, social, artistic, and personal/persuasive [20]. Following the idea to make information available to casual users, some studies focus on the visualization of urban data in public displays. In [82], the authors present an analysis of installations (i.e., public visualizations situated in the real world) related to urban data, criticizing the lack of a more complex view of the city dynamics in the visualization, often focusing on a single attribute (e.g., energy and water use). In the Reveal-it! project, the authors, presenting a study on energy consumption, prove that social visualizations available in public settings are able to increase citizens’ awareness by showing underlying patterns in the data [81, 83]. A different approach, with a similar goal, is presented in [84], in which authors detail the design and evaluation of a kit that empowers citizens to visualize data in wireless small e-link displays, situated in visible space (i.e., a shop showcase). Focusing on air quality monitoring, Hsu et al. present a system that combines animated smoke images, air quality data, crowdsourced smell reports, and wind data to empower the community to support policymaking [85]. Data related to the implications of paperless initiatives in terms of paper waste avoided and benefits for the environment have been presented in two infographics designed to exploit different styles and techniques, to extract insights related to the design of infographics to increase awareness about sustainability issues [86].

Lately, Augmented reality (AR) and Virtual Reality (VR) have been exploited to visualize data related to the environment directly on the smartphone [22]. In [27], the authors present the potential of VR for data representations, arguing that, by specifying information-dense virtual environments in relation to the capacity of human ecological perceptions, there is an opportunity to develop new approaches to exploring patterns, anomalies, and connections within big data. In the mobile ecosystem, USC AiR is a mobile application that translates the air quality sensor feeds from an IoT infrastructure, into augmented reality visualizations [87].

Indeed, the new big data paradigm brings up new challenges as well as new opportunities in the field of data visualization [88–90].

All the presented studies reveal the interest in making sense of urban data, engaging casual users. Drawing from their outcomes, our approach aims to investigate the consequences to provide communities with visualizations related to bid data, and in particular, to tourism flows, strongly impacting the sustainability of tourist destinations, following the strategy to provide simple and meaningful visualizations that can be overlaid to create knowledge on a specific issue.

## The platform walkthrough

As anticipated, we designed and deployed a platform composed of two systems:

- Beanstalk: a community-based infrastructure designed to collect and make data related to tourism flows and different sources of social networks and environmental parameters open and accessible [8, 60].
- ViTFlow: a rich interactive web-based system that takes the Beanstalk authors’ effort further by making the information available to the internet general public, creating opportunities for reflections and awareness about the hidden impacts of tourism flows among locals and visitors.

**Fig. 1:**
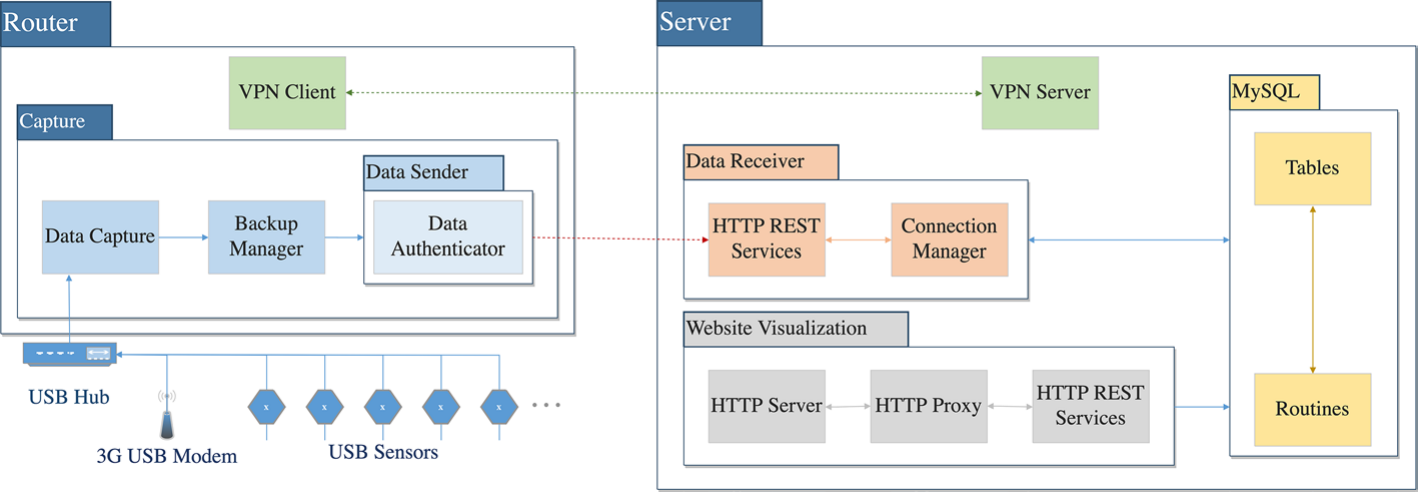
The passive Wi-Fi tracking architecture

**Fig. 2:**
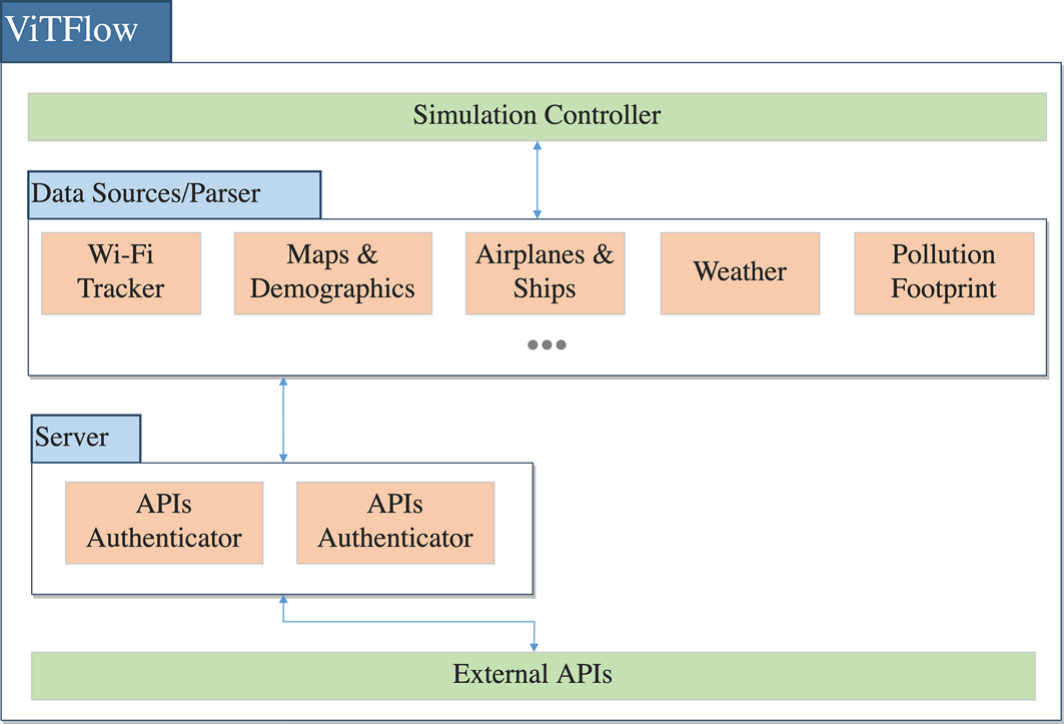
The ViTFlow architecture

To accomplish this goal, we designed a modular system composed of different hardware/software components. In particular, three components can be highlighted: (i) the passive Wi-Fi infrastructure, integrated with USB sensors, to gather data about air quality, weather condition, and noise; (ii) the back-end application, including the database and exposing APIs to obtain aggregated and structured data; (iii) the ViTFlow rich web application, visualizing spatio-temporal data, obtained from different data sources. Figure 1 shows the modular architecture of the passive Wi-Fi tracking infrastructure, the back-end application, and how the different components interact with each other. Conversely, Fig. 2 displays the main constituents of the web interface for visualizing urban data. We designed the architecture following a modular approach, to make it extensible to other data/layers. In the next subsections, we focus on describing the low-cost infrastructure to gather urban data , and the rich map-based interface to visualize the data .

### Beanstalk: a low-cost infrastructure to gather urban data

The Beanstalk infrastructure was deployed at more than 80 points of interest in Madeira islands which provides a unique testbed comprising 270 000 inhabitants (that makes it the third metropolitan area in Portugal) with more than 1,3 million yearly visitors. Madeira Islands are part of the Natura 2000, a network of nature protection areas in the territory of the European Union which despite the high density of population covers 2/3 of the territory. This combination of a historical touristic region, hosting both a major metropolitan area and a particularly rich nature reserve, provides a unique testbed for studying these phenomena from a big data perspective. After an initial test phase, with 14 routers installed at the airport, port, and several squares in the city capital, the system was expanded to more than 80 routers placed nearby the main points of interest (POIs) covering the more popular tourist attractions of the two islands (Madeira and Porto Santo).

It is important to notice that the routers were installed not only in locations owned by the tourism board and the local government, but also by citizens, inviting them to install the system outside of restaurants, bars, hotels, and so on, simply engaging the local owners in participating to expand the infrastructure. In fact, owners were attracted by the idea to be part of the system for collecting data, with the final aim of informing the communities about dynamics that impact the sustainability of the islands. In each POI, internet access was guaranteed by the receiving entity. Moreover, in collaboration with the main public bus company in Madeira, we augmented the Beanstalk infrastructure with additional routers placed in 20 buses covering the high-frequency routes in Funchal, the capital city, expanding the coverage of the network with mobile stations.

The routers are used to capture the probe requests, which are special frames sent by devices to discovery networks. The frame is broadcasted from the device to the networks reachable in a specific range and it contains the ID of the device making the request (MAC address). To avoid issues related to privacy concerns, the MAC address is stored as a cryptographic hash, together with a location ID of the router, and a timestamp (see [60] for more details). This allows to locate a device in space and time, to obtain a snapshot of the connected devices in each location, and to compute the more common mobility paths and mobility flows around the island. Another interesting data encapsulated in the probe request is the SSIDs history, a list with the names of the saved networks on the device. This list provides an additional method for profiling users, as described in [49]. We used the SSID profiling and a clustering algorithm to classify devices as locals or tourists, as presented in [60].

Since the routers have a USB port, we used these as nodes of a hub to connect multiple sensors for environmental monitoring. With the intent to make the system modular, sensors with a USB interface, or that could be read through intermediate hardware, were chosen. At this state, the sensed data are: particles (PM2.5, PM10), ozone (O$_{3}$), carbon dioxide (CO$_{2}$), sulphur dioxide (SO$_{2}$), nitrogen dioxide (NO$_{2}$), carbon monoxide (CO), nitrogen oxide (NO), temperature, humidity, air pressure, and noise.

Over a period of four years, the Beanstalk infrastructure collected mobility data on more than three million unique devices, resulting in a total of 480 million data points. To the best of our knowledge, the Beanstalk dataset represents a unique case study, in terms of size and duration, both compared to data collected over commercial systems, and data gathered in different academic studies. For more details about the Beanstalk infrastructure and the dataset analysis, see [8, 60].

### ViTFlow: a rich application for data visualization

To make sense of the high variety of multi-sourced data, we designed and implemented ViTFlow (Visualising Tourism Flows). The application was built using standard Web Technologies, mainly HTML5, CSS3, Javascript and NodeJS, and SVGs. ViTFlow is composed of several visualizations (layers) that display different animations informed by the collected data. As displayed in Fig. 2, when a user requests a layer, ViTFlow launches a Node.js application that establishes a connection with the API providers and returns the data requested to the client. This data is then processed from the API provider entity, according to the visualization requested, and is returned to the client in a JSON file. Each visualization is declared in the system as a module that has its own data processing and animation. New visualizations can be created and added to the application, following the defined template, so as new APIs, other than the ones in place, can be supported, allowing new data to be represented.

Regarding the rendering of the visualizations, ViTFlow uses the SVG transformation techniques available on modern browsers, through the primitives provided by the D3.js data visualization framework^1^. The visualizations, each one originated from a data source, are all centered in a digital abstract map of the islands and represented on the sub-regions themselves or are layered on top of them. The map, used as a base for the visualizations, is the official Administrative Map of Portugal (CAOP 2019^2^) originally provided in a shapefile format. This file is then converted to geoJSON and contains information regarding each administrative district, including its borders. This file is rendered using the map utilities provided by D3.js, which are used throughout the application to transform geographic coordinates into absolute positions (x,y) on the screen. With the absolute positions, we can perform SVGs transformations such as plotting points onto the map or transitioning an object along a path. The map can be zoomed using the scroll up/down movement, navigated by clicking and moving the cursor, and district information can be accessed by selecting the respective section on the map. Certain visualizations also make use of the mouseover to display extra information, such as the number of tourists in a city or the specific average temperature.

**Fig. 3:**
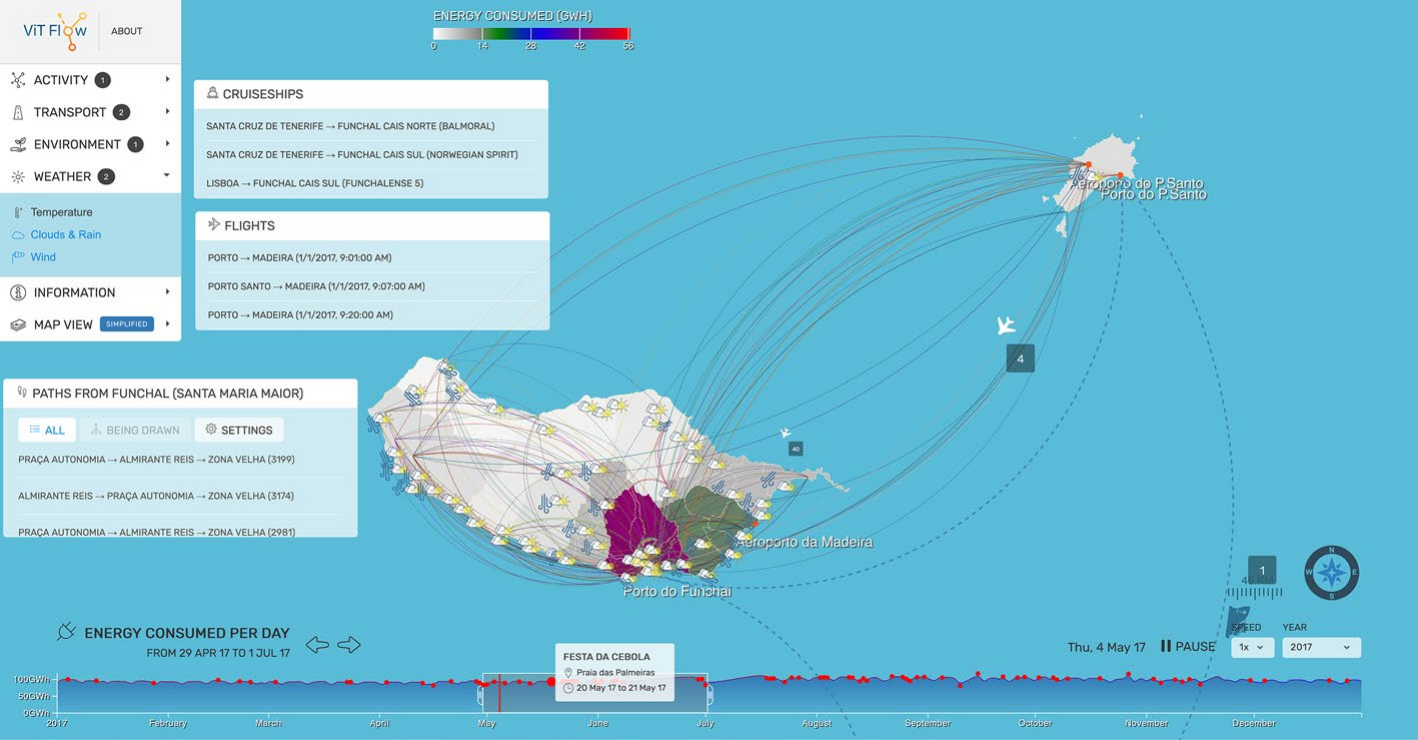
A screenshot of the ViTFlow rich interface where different datasets/layers are visualized in a given time

Figure 3 presents a screenshot of the ViTFlow rich interface. On the top left corner of the interface, there is a navigation menu, which allows the selection of multiple layers, from different categories, such as: Movements, Transportation, Environment, Weather, Information, and Map View. In Fig. 3, six layers have been activated and visualized: one in the Activity category; two in the Transport category; one in the Environment category; and two in the Weather category. Visualizations with historical data have a timeline graph (depicted on the button of Fig. 3) that allows users to select the data referring to one year or to smaller subsets of time, using a brushing technique that allows selecting a specific period of time (in Fig. 3, from May to July). The timeline graph also provides the events that occurred, daily, represented as red dots. Each dot can be selected to provide specific information about the event, such as the name and the location (in Fig. 3, the *Festa da Cebola* event is selected). Events are collected by experts, using official touristic sources, and can be updated using a crowdsourcing approach by the users who can interact with the back-end application. The timeline graph has a red bar that indicates the current time of the visualization. With the bar progressing in the timeline (daily), the data on the screen is updated. The timeline graph is also important to visualize, at glance, the aggregated data (e.g., the total amount of CO$_{2}$ emissions in all the Madeira Islands in a given day, or, as in Fig. 3, the energy consumed per day) distributed in the timeline.

It is worth noticing that multiple visualizations can be activated at the same time. In such a case, the system synchronizes the timing of all the visualizations to produce a single animation with all the data. Each specific timeline graph can be consulted using the arrows presented next to the title of the layer, moving from layer to layer (in Fig. 3, the “Energy consumed per day” layer has been selected). Using the arrows, the user can give the focus to a specific layer, maintaining the other ones in the background. Selecting the visualization (and time window), the system starts playing an animation, recreating the data changes on the map chronologically. The user can also manipulate the animation progress using the speed control and the pause/start button (visible at the bottom-right of Fig. 3).

With the purpose of turning this into a public display for the citizens to interact with it, ViTFlow supports integration with certain keys on the keyboard to navigate through the menus, activating/deactivating visualizations, and performing actions on the application. A gamepad integration is also available to navigate the menus through the mapping of the keyboard shortcuts into the gamepad with an intermediate software, aimed to attract users’ attention in public spaces, without the need of using a special/touch screen display.

## ViTFlow in detail

ViTFlow provides communities with different visualizations (layers) in order to make sense of diverse variables related to tourism flows and urban data. In particular, all the categories of variables and the included layers are presented in Table 1.

**Table 1:** The main categories and layers provided in ViTFlow

Main category	Layers
Activity	Movements
	Common Paths
	Demography
	Tourists
Transport	Airplanes
	Cruise ships
Environmental	Energy consumption
	CO2 emissions
Weather	Temperature
	Clouds and Rain
	Wind
Information	Routers status
Map view	Simplified
	Humanitarian
	Satellite
	Street

In the following sub-sections, we describe each data source and layer associated with it, grouping the layers based on the graphical visualization we decided to design and implement (i.e., heat map, pie, etc.) to make the presented information more appealing to citizens. We will focus on the main categories: Activity, Transport, Environmental, and Weather.

### Heat map visualizations

Heat maps were used to visualize the distribution of a specific variable in each region. Three layers made use of heat maps: Tourists, Energy Consumption and Temperature.

#### Activities–tourists

**Fig. 4:**
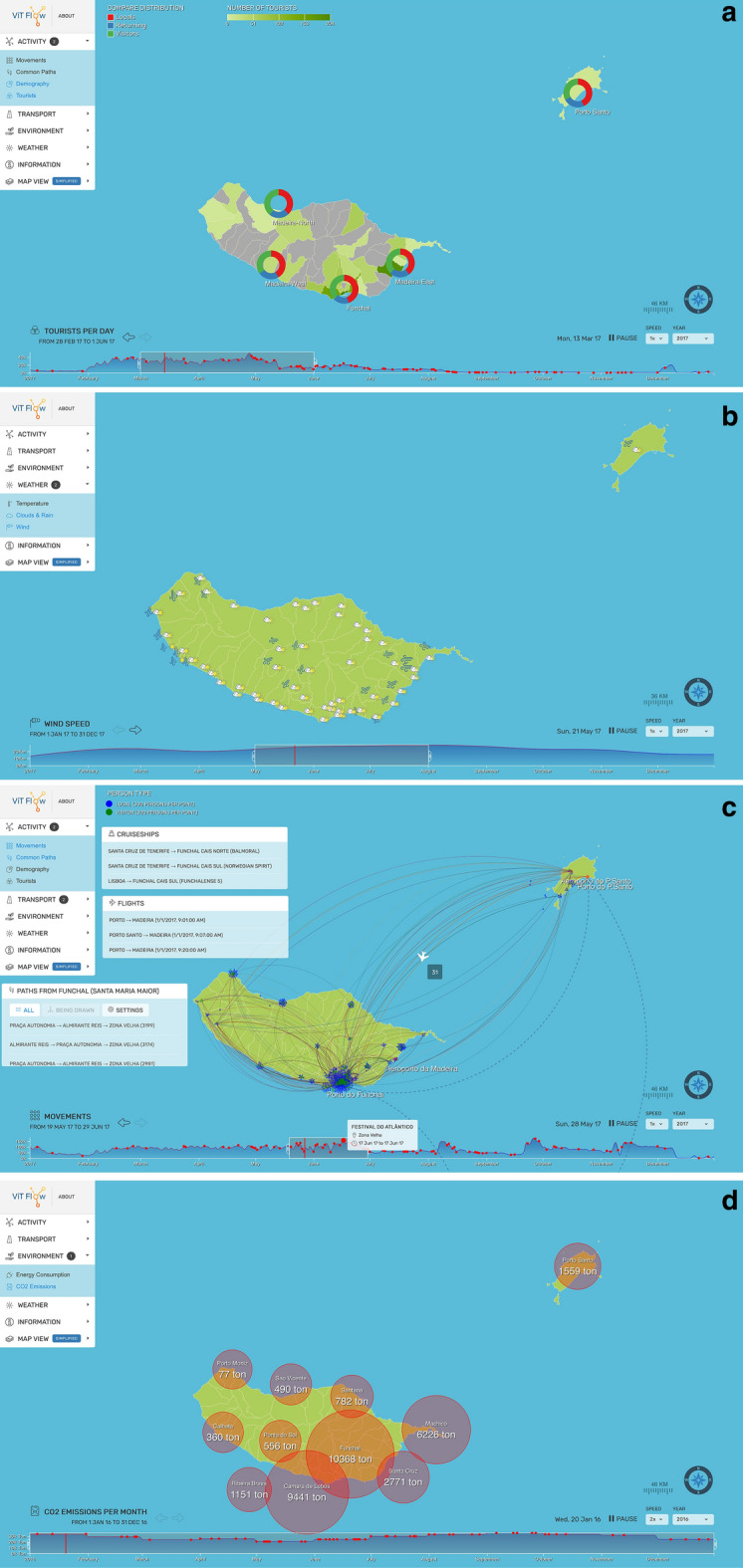
Some screenshots of the ViTFlow rich interface displaying different data visualization strategies

The Tourists visualization shows a heat map with the distribution of tourists across the island, which allows users to quickly see how many tourists any region has at any given time, and how the data changed historically (as shown in Fig. 4a). As Beanstalk continues to collect data, it will also be possible to compare tourist flows across different years, to understand trends. Users, observing the timeline graph can immediately see the island most visited period of the year, since it presents the aggregate number of tourists on the island.

#### Environment–energy consumption

The layer Energy Consumption exploits a heat map to relate the energy consumption (in GWH) in the main Madeira Islands geographic areas (as shown in Fig. 3). Selecting a specific area, it is also possible to obtain the details about the estimated origin (e.g., thermal, wind, solar, etc.). The data is provided by official energy statistics^3^. Users can explore this layer to understand the consumption of energy in the islands, how it is generated, and the overall sustainability impact.

#### Weather–temperature

The Temperature layer uses a heat map to visualize the degree in the island in each area, ranging between 5 °C and 30 °C. The resulting degree value is the average of the different temperatures sensed in the area in a given month. The users, mouse-hovering the area can access the actual temperature. These data have been gathered by the API exposed by Observatório Oceânico da Madeira^4^.

### Icons reflecting specific values

Dynamically mutating icons represent different weather conditions. The icons change based on the represented condition. For instance, the clouds icon becomes more transparent depending on the clouds density, while the wind icon changes its orientation to indicate the wind direction.

#### Weather–clouds and Rain

To present the percentage of clouds and rain, we used clouds and sun icons (as shown in Fig. 4b). We decided to use also the sun (instead of just clouds), to emphasize the presence of nice weather (instead of just removing the clouds icon). For this reason, we played with the colour saturation and transparency to display the different percentages of cloudiness and precipitation, and sun. The percentages are accessible through mouse-hovering on a specific area in proximity of the icon.

#### Weather–wind

The wind is a very important factor on the island, influencing the landing of airplanes in the airport (and the island mobility). That is why we opted to include it in the visualization. In ViTFlow, the wind is represented by a dynamic icon that changes size according to the wind speed, and orientation based on the wind direction (as shown in Fig. 4b). By selecting the wind icon, it is possible to see the detailed speed and orientation values.

### Dynamic trajectories and paths

Dynamic trajectories from origin to destination are used to animate visualizations in four ViTFlow Layers: Movement, Common paths, Airplanes, and Cruise ships. Figure 4c depicts the four layers activated.

#### Activities–common path

The Common Path layer allows users to select a city and see the most common paths that start in that city. Each drawn path (a line) has a specific color and its thickness depends on the estimated number of people who followed that path. The number is calculated using the average considering all the collected data by the Beanstalk infrastructure. To start the animation, the user selects an area in the map (in Fig. 4c, *Funchal - Santa Maria Maior*), that acts as the origin of the common paths. Moreover, an external panel pops up to detail the common paths that are drawn in the digital map, making explicit all the regions followed from origin to destination. The user can also choose if visualizing the paths one by one or all together.

#### Activities–movements

The Movements layer presents information about mobility flows in the islands, differentiating tourists from citizens through coloured trajectories, drawn by small dots (blue for locals and green for visitors), as shown in Fig. 4c. Each dot represents a specific number of users (as defined in the legend). In particular, it displays the distribution of people on the island and the movements they make across time, data gathered thanks to the Beanstalk infrastructure (more details about the dataset analysis are available in [60]). To display this information, we exploited the idea of showing the flows of people using dots that float on the digital maps and move from origin to destination. The data is gathered by our passive Wi-Fi tracking infrastructure and made available using the back-end API. Essentially, we display the number of devices detected in a specific area (router) in a day and the movement of these devices around the island in the following days.

#### Transportation–airplanes

The Airplanes layer displays the number of planes that land in the airport on a given day (as shown in Fig. 4c). The airplanes are drawing so as to simulate two origin locations, differentiating the ones coming from the smaller island (Porto Santo) and from the mainland, and foreign destinations. Besides the map-based layer, this visualization makes visible also a panel detailing the information of the planes landing in Madeira, such as origin, date, and time. To obtain this data, the system queries the API provided by the local airport management company (ANA^5^).

#### Transportation–cruiseships

This layer works similarly to the airplanes (including the external panel with details the information, as shown in Fig. 4c. In this case, it visualizes two trajectories: arriving in Funchal (from Porto Santo, the smaller island), arriving in Funchal (from elsewhere including the mainland). The dataset is provided by the port authority of Madeira (APRAM^6^). This information is relevant not only considering the amount of CO$_{2}$, but also other dynamic variables. In fact, the arrival of a cruise ship pours out a significant number of visitors, influencing the urban mobility and the overall number of tourists.

### Pie charts

We decided to use pie charts to provide static information, estimated as an average of the gathered data.

#### Activities–demography

To visualize the ratio between residents and visitors in different areas, users can activate the Demography layer. Using a pie chart, we present the amount (in percentage) of tourists (the green part) and residents (the red part) aggregating the data considering the main areas in the island (as visible in Fig. 4a). With the term tourists and residents, it is intended the number of devices sensed by the Beanstalk infrastructure and then classified using the SSID profiling technique [60]. The values are calculated considering all the gathered data. At a glance, the user can see the ratio between residents and visitors, and, on mouse-hovering a specific area, it is possible also to learn the exact number of the two categories of users.

### Dynamic bubbles chart

To show information strictly related to the footprint calculation, we opted for displaying floating bubbles, located on the digital maps.

#### Environment–CO$_{2}$ emission

An important layer, considering environmental sustainability, is the one visualizing CO$_{2}$ emissions. As anticipated, we used bubbles, floating on the top of the related region (see Fig. 4d). The bubble size represents the amount of CO$_{2}$ emission (in TON) in each area in a specific month (it is calculated and aggregated month by month). Data is gathered both using the API provided by the Beanstalk back-end application and from PORDATA,^7^ an external source.

## Evaluating ViTFlow

In order to validate ViTFlow against our research aims we performed two field studies to understand: (i) how people react to tourism flow visualizations and their environmental impact; (ii) how much people feel engaged in using the system and discovering complex sustainability-related dynamics.

All participants engaged in the two experiments were informed about the evaluation protocol before starting the data collection. The participation was voluntary, and all participants had the right to comply with or refuse participation, considering also details about the data storage and analysis (accordingly with European General Data Protection Regulation).

### A preliminary field study: local public event

To collect the users’ reactions and feedback we performed a preliminary field study during a local public event, shadowing the users and taking notes of their interactions with the system and aloud comments. The study was conducted installing the public display, using a 24-inch screen and a gamepad, in an informal environment close to a popular pedestrian street in Funchal. At the end of the interaction, we asked users a few general questions to know their age, nationality, current residence, and a couple of questions related to the notes we took observing their interaction with ViTFlow.

#### Participants

During the three hours session, 23 users approached our system (4 tourists and 19 residents). Participants (9 females and 14 males) ranged from 19 to 71 years of age. On average, each interaction lasted 14 minutes. Users approached the public interface mostly in groups (couples or groups of friends).

#### Insights

All the participants enjoyed the Transport layers graphic, but some of them (7 out of 23) found it difficult to associate the represented information related to the number of airplanes and cruise ships arriving on the island with the data related to the origin and destination. A man living in Madeira, when saw the airplanes landing on the island, exclaimed, joking with her partner: “What is happening? Who is invading the island?”. During the interview, he revealed to us that he works in the airport, but seeing the exact number of airplanes landing on the island, day by day, was “impressive”.

Besides the Transport visualization that attracted the curiosity of all the users, tourists showed more interest in the Weather visualizations, while residents in the Activity layers (i.e., mobility patterns and demography). Some users (7 out of 23) checked all the visualizations before leaving the interaction. This means, that, since people approached the installation in groups of 2 or 3 people, almost all groups saw all the visualizations. It was also possible to notice that, in each group, only one person was keeping control of the interaction for all the session, navigating the interface also following the indication of the friends/partner.

A relevant result was related to the use of different visualizations to provide aggregated knowledge. In fact, once users discovered the possibility to overlap layers, they liked to activate and mix different visualizations. Interesting was noticing that they were not only interested in the graphic aspect of the resulting visualization, but they looked for correlations between the presented data (such as the number of events and flights in a specific month, and demography of tourists and citizens and weather condition in a specific area). Users started discussing with each other about the correlations they founded. Accordingly, a typical starting sentence was “Look here, how many”. Drawing on these insights we can affirm that the visualizations functioned as a discussion tool, stimulating conversations between the groups/couple, and confirming the interests of residents and tourists in this kind of information and visualizations. Three users, during the short final interview, told us that it would be really interesting to see also the data related to energy consumption (a complex issue in the island) and CO$_{2}$ emissions, so as to have a more complete view of the sustainability of the island.

This preliminary field study was conducted in a public and crowded space and with our constant presence close to users. To avoid biases due to such a context, we decided to carry out another study to collect quantitative data, letting the user free to explore ViTFlow, as long as they enjoyed it, in their homes or preferred locations and devices.

### Second study: online surfing and survey

We improved our system considering the findings of the preliminary study and we decided to evaluate it using an online survey, to collect quantitative data about the sessions and interactions, letting the users free to interact with the system as long as they want. We designed the second study to obtain quantitative values to better understand the users’ experience and the system performance as a tool to inform users (as explained in the next subsections). We also integrated new data sources, such as energy consumption and CO$_{2}$ emissions, as suggested by some users during the first study, to facilitate both communities of locals and visitors in developing a more informed opinion on their sustainable/ecological footprint. We created the questionnaire focusing on the local communities, with the intent to measure, from a quantitative point of view, the ability of ViTFlow to inform people about tourism dynamics impacting sustainability. We decided to focus this study on residents for two reasons: first, we wanted to ask questions strictly related to Madeira with the intent to measure if the system can provide new information (for tourists, this statement is biased by the fact that they don’t own previous knowledge of the island and its dynamics); second, for the first study it was clear that the residents are more interested in understanding the visualized data and the phenomena resulting from the meaningful representations.

#### The survey

The survey was deployed in the Portuguese languages, and was structured as follows: a first section with general/contextual questions (11 items); a second one including specific questions about Madeira (9 items); a third one in which we ask users to interact with the web system (free interaction); finally, the last part was related to questions concerning the interaction with the system (14 items), including the same questions of the second section together with a few questions about the ViTFlow interface. 20 of 23 items were multi-choice questions, including simple decision values or a five-value Likert scale. We also included three open questions to obtain qualitative data and feedback: in one item we asked to write a definition of sustainably; another one was related to writing sustainability activities carry out in the islands; and, the last one was for open feedback and comments about ViTFlow. At the beginning of the survey, we provided a short description of the project, stressing the fact that data are collected anonymously and only for research purposes (accordingly with the EU General Data Protection Regulation). We distributed the survey among local people, using word of mouth, and we incentivized participants to share with family and friends. Together with the survey, we instrumented our web application with software (Hotjar^8^) to log interactions (i.e., clicks) and record user’s sessions, to know the duration of each interaction and the more selected visualizations.

#### Participants

**Fig. 5:**
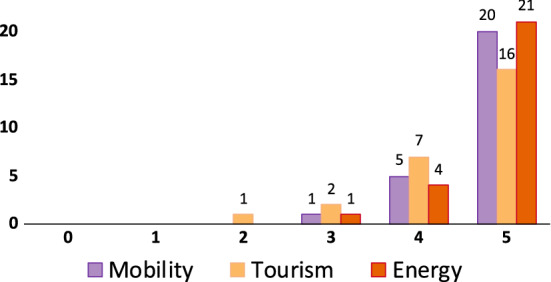
Participants’ consideration about the relation between mobility/tourism/energy and sustainability

We obtained 26 responses. The age of participants ranged from 24 to 54, with 11 (42.3%) males and 15 (57.7%) females. Almost all users were locals, except for two users: one from the mainland and another from an emigration country (Venezuela). Our survey probed users about their level of education to understand their background: 2 users had a Ph.D, 7 had a Master’s degree, 13 a Bachelor’s degree, and 4 a high school degree. Considering the occupation: 9 participants worked on education (4) and research (5) sectors; 5 worked on the IT sector; 4 in energy; 3 in transportation; and the rest of the participants cover diverse areas such as tourism, health, farming/agriculture, restaurant/food and drink services, journalism. Only one user didn’t specify his/her occupation. In order to understand the users’ existing level of awareness on sustainability issues, we asked participants to define “sustainability” in an open question. Almost all participants (23/26) provided a definition, and the answers were surprisingly thoughtful and articulated. For instance, one participant wrote: “Sustainability is part of what each person does in their day to day life to build a better world”. Instead, another one claimed: “Behaviors that do not harm or damage the environment, including flora and fauna”. Interesting to notice that several responses included the terms “future” and/or “long term”. We also asked participants to express, using a five-value Likert scale, how much they consider mobility/tourism/energy an important factor to keep into account to improve the sustainability of the island. The results are shown in Fig. 5, indicating tourism as the perceived less impacting factor on sustainability.

#### ViTFlow evaluations

As anticipated, the second and fourth sections in the survey were aimed at measuring the level of information conveyed by the visualizations and understood by the users. We asked participants to answer questions related to variables influencing the islands (i) mobility (such as, “In your opinion, which is the season with more cruise ships docking in Madeira?”), (ii) tourism (e.g., “How much do you think the weather influences the arrival of tourists in the island?”), and (iii) sustainability (for instance, “Do you agree with this sentence: The amount of energy consumed in Machico is, in average, more than the one consumed in Camera de Lobos”). The questions are referring to common knowledge/insights. We wanted to measure if, after interacting with the system, users changed their answers according to the visualizations. To assess the significance of the experimental results from a statistical point of view, we carried out a Paired Sample t-test analysis for each question item of section 2 (comparing the data with the answers of the same items in section 4, post-interaction) in the survey. As null hypotheses, we defined the following: H0: The user did not increase her/his awareness considering the information visualized in ViTFlow, concerning mobility, tourism, and sustainability.

Our results strongly validate (with p < 0.05, one tail) the alternative hypothesis (i.e., the user did increase her/his awareness) only for three questions of the survey, and weakly confirm it (considering p < 0.10, one tail) for 2 questions. The results show, as expected, that users’ previous knowledge about the variables strongly influences their responses, and this is particularly true in our sample where different people work in sustainability-related fields (as tourism, transportation, and energy). Consequently, their previous knowledge is visible in the results. Nonetheless, analyzing the answers in detail, it is possible to observe that the majority of users who changed the answer after interacting with ViTFlow, tended to the right answer.

**Fig. 6:**
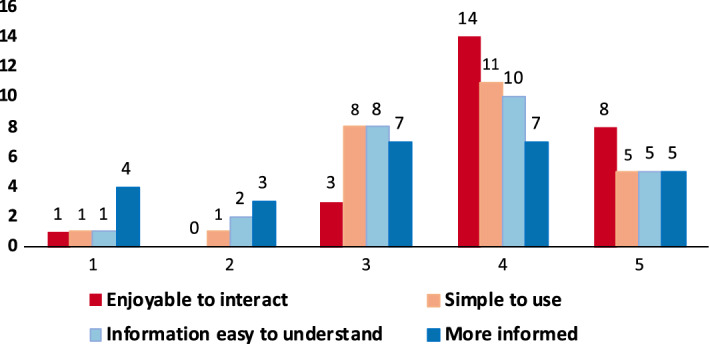
Participants’ opinion about ViTFlow

The last part of the survey (4 items) collected data about the ViTFlow users’ experience. The results are presented in Fig. 6. One participant didn’t enjoy the system at all, answering “Not at all” to all the questions. Analyzing the session data, it also possible to notice that he/she interacted with the system less than 3 minutes due to a problem with the loading of the data. Nevertheless, in general, users enjoyed the system (22 positive, 1 negative, 3 neutral answers) and found it simple to use (16 positive, 2 negative, 8 neutral answers). Conversely, some users did not find the information very easy to understand (3 negative, 8 neutral, 15 positive answers) or felt more informed after interacting with ViTFlow (7 negative, 7 neutral, 12 positive answers). This result is also confirmed by the statistical analysis we performed.

#### Sessions analysis

From the analyses of the session logs we can state that, on average, users interacted with ViTFlow for 8:15 minutes (considering an active interaction) with a standard deviation of 3:13. The short interaction lasted less than 3 minutes, and in the final comment in the survey, the participant wrote that the system took too much in loading data and he/she just decided to close the application. The longer active session lasted almost 16 minutes. Looking at the heat map of the clicks on the main page, clicks are concentrated in the menu area, as expected. Moreover, it is interesting to notice that all areas compared with the others present a similar number of clicks. This can be explained considering the fact that users were curious/interested to explore all the visualizations in our ViTFlow interface.

## Discussion

The two studies let us better evaluate the impact of ViTFlow as a tool to stimulate discussion and awareness on the sustainability of the Madeira islands, in terms of mobility flows and transportations, CO$_{2}$ emissions and energy consumptions.

The first study confirmed our aim to design a system able to stimulate reflections on the visualized data. In fact, users discussed the data, questioning and looking for confirmation about the meaning of the data visualized. It is possible that this discussion was facilitated by the fact that users approached the system in groups. Another positive outcome was to see that the users not only enjoyed the idea to have different layers that can be activated/deactivated, but they also understood the relevance to this mechanism to obtain a global overview of a specific phenomenon involving different variables. This was particularly true for citizens who demonstrated curiosity and interest in understanding and making sense of data related to the place where they live.

The second study was more focused on collecting quantitative data to measure the impact of the system and evaluating ViTFlow. Addressing the questionnaire to residents, we obtained interesting insights that can be summarized as follow. Considering the interaction with the system, a strong majority of users enjoyed the system (22 out of 26) and found it simple to use, and 15 found the information very easy to understand. These results are a confirmation of our design choices aimed to make sense in an easy way to high volumes of data. Regarding the impact of the system in informing the users, the majority of users (14 out of 26) didn’t answer positively to the question stressing this measure. This result is also confirmed by the statistical analysis we performed. The explanation can be found in examining in detail the sample of users who participated in the study: most of them are working in the sustainability-related field. For this reason, this measure needs to be investigated in a different scenario, enlarging the sample of users to better represent the Madeira population. This will also allow overcoming another limitation of this study, related to the number of participants (sample size) engaged in the two experiments (23 in the preliminary field study and 26 in the second study). These sample sizes can still provide valid results, as discussed in [91], but, indeed, engaging a greater number of participants can strengthen the obtained results, reducing the biases.

To make the system available to all the casual users, and overcome the issues related to the number of participants and their background, we are planning to install it in different strategic points of interest across the Madeira island, with the intent to stimulate discussion based on the data (how it happened during the first study). In doing that, it is important to keep into account that, while interactive displays have a high potential to engage passers-by, they frequently go unnoticed and unused [92, 93], confirming the so called ‘display blindness’ effect [94]. By situating our display in strategic locations, we intend to mitigate this issue, exposing users to the ViTFlow at a time when they will be inclined to interact with it. Moreover, our solution aims to overcome the ‘interaction blindness’ [95] that often plagues public displays by providing users with animated visualizations that encourage non-linear exploration. The use of a gamepad as a form of a physical input device can limit the ‘affordance blindness’ issue, defined as the inability to understand the interaction modalities of a public display [96].

Concluding, the outcome of this research work confirms the interest of casual users in understanding and becoming informed about urban and environmental big data. This is particularly true for citizens, since these data have an impact on (and are impacted by) their daily life. In this context becomes fundamental to make big data easy to understand and open/accessible to casual citizens (as discussed in recent studies [84, 85]). The issue becomes even more urgent considering (i) data impacting sustainability, and (ii) the context of a touristic destination, where data are augmented by a relevant number of tourists (with 270.000 inhabitants, Madeira attracts more than 1,3 million tourists per year), making the category of urban and environmental data affected by tourism, even less “visible” to citizens.

## Conclusion

In this paper, we present the design and implementation of an interactive platform that exploits a pervasive sensing infrastructure to collect multi-sourced urban data and mobility flows (named Beanstalk) and visualizes these data in a rich map-based web interface to make sense to gather big data (named ViTFlow). Beanstalk was been deployed thanks to the involvement of citizens to collect urban data about tourist flows and environmental conditions, thanks to a pervasive IoT and sensors infrastructure. Moreover, we designed ViTFlow with the goal of presenting data, often imperceptible, to the interested communities of locals and tourists, to make them more informed about dynamics affecting the sustainability of the Madeira Islands, such as mobility patterns, tourism flows, energy consumption, and CO$_{2}$ emission. We evaluated our system with two experiments that provided interesting insights to demonstrate, through both the analysis of qualitative and quantitative data, that our system can be exploited to benefit citizens and communities, in order to enhance their awareness. Drawing on the obtained findings, as future work, we plan to develop ViTFlow in public display in strategic points around the islands to potentially reach all the interested communities. Concluding, the development of our case study let us reflect on two interesting challenges that need to be better investigated related to citizens participation and appropriation of the infrastructure, to develop user-centered and transparent intelligent urban environments: (i) stimulate the participation of citizens in order to become providers of big data, enlarging the pervasive infrastructure; (ii) foster citizens participation to make sense to big data towards a sustainable development.

## Data Availability

The datasets analysed during the current study (i.e., collected during the two studies) are available from the corresponding author on reasonable request. Nonetheless, restrictions apply to the availability of data visualized in web system, which were used under license for the current study, and so are not publicly available.

## Abbreviations

CO: Carbon monoxide
CO$_{2}$: carbon dioxide
IoT: Internet of Things
N$$_{2}$$O: Nitrous oxide
NO: Nnitrogen oxide
O$$_{2}$$: Ozone
PM: Particulate matter
POI: Point of interest
ViTFlow: Visualising Tourism Flows

## Footnotes

1 https://d3js.org.

2 https://www.dgterritorio.gov.pt/cartografia/cartografia-tematica/caop.

3 http://estatistica.madeira.gov.pt

4 http://oom.arditi.pt

5 https://www.ana.pt/.

6 http://www.apram.pt/.

7 https://www.pordata.pt/.

8 https://www.hotjar.com.

## References

[1] Cohen SA, Higham JE, Stefan G, Peeters P. Understanding and governing sustainable tourism mobility: Psychological and behavioural approaches. 2014.

[2] McKercher B (1993). The unrecognized threat to tourism: can tourism survive ‘sustainability’?. Tourism management.

[3] Verbeek D, Mommaas H (2008). Transitions to sustainable tourism mobility: The social practices approach. J Sustain Tour.

[4] Garrigos-Simon FJ, Narangajavana-Kaosiri Y, Lengua-Lengua I (2018). Tourism and sustainability: A bibliometric and visualization analysis. Sustainability.

[5] Bibri SE (2019). The anatomy of the data-driven smart sustainable city: instrumentation, datafication, computerization and related applications. J Big Data.

[6] Bibri SE (2019). On the sustainability of smart and smarter cities in the era of big data: an interdisciplinary and transdisciplinary literature review. J Big Data.

[7] Maeda TN, Shiode N, Zhong C, Mori J, Sakimoto T (2019). Detecting and understanding urban changes through decomposing the numbers of visitors’ arrivals using human mobility data. J Big Data.

[8] Nunes N, Ribeiro M, Prandi C, Nisi V. Beanstalk: a community based passive wi-fi tracking system for analysing tourism dynamics. In: Proceedings of the ACM SIGCHI Symposium on Engineering Interactive Computing Systems, 2017;pp. 93–98.

[9] Redin D, Vilela D, Nunes N, Ribeiro M, Prandi C. Vitflow: a platform to visualize tourists flows in a rich interactive map-based interface. In: 2017 Sustainable Internet and ICT for Sustainability (SustainIT), 2017;pp. 1–2. IEEE

[10] Boieiro M, Aguiar AF, Rego C, Borges PA, Serrano AR. The biodiversity of terrestrial arthropods in madeira and selvagens archipelagos. Revista IDE@-SEA 6, 2015;1–20.

[11] Zheng Y, Zhang L, Xie X, Ma W-Y. Mining interesting locations and travel sequences from gps trajectories. In: Proceedings of the 18th International Conference on World Wide Web, 2009;pp. 791–800.

[12] Zheng W, Huang X, Li Y (2017). Understanding the tourist mobility using gps: Where is the next place?. Tour Manag.

[13] Gabrielli L, Rinzivillo S, Ronzano F, Villatoro D. From tweets to semantic trajectories: mining anomalous urban mobility patterns. In: International Workshop on Citizen in Sensor Networks, 2013. p. 26–35. Springer

[14] Chen Y-Y, Cheng A-J, Hsu WH (2013). Travel recommendation by mining people attributes and travel group types from community-contributed photos. IEEE Trans Multimedia.

[15] Bonné B, Barzan A, Quax P, Lamotte W. Wifipi: Involuntary tracking of visitors at mass events. In: 2013 IEEE 14th International Symposium On” A World of Wireless, Mobile and Multimedia Networks”(WoWMoM), 2013. p. 1–6.

[16] Ruiz-Ruiz AJ, Blunck H, Prentow TS, Stisen A, Kjærgaard MB. Analysis methods for extracting knowledge from large-scale wifi monitoring to inform building facility planning. In: 2014 IEEE International Conference on Pervasive Computing and Communications (PerCom), 2014. p. 130–138.

[17] Kjærgaard MB, Wirz M, Roggen D, Tröster G. Mobile sensing of pedestrian flocks in indoor environments using wifi signals. In: 2012 IEEE International Conference on Pervasive Computing and Communications, 2012.p. 95–102.

[18] Nikzad N, Verma N, Ziftci C, Bales E, Quick N, Zappi P, Patrick K, Dasgupta S, Krueger I, Rosing T.Š. *et al*. Citisense: improving geospatial environmental assessment of air quality using a wireless personal exposure monitoring system. In: Proceedings of the Conference on Wireless Health, 2012. p. 1–8.

[19] Dutta P, Aoki PM, Kumar N, Mainwaring A, Myers C, Willett W, Woodruff A. Common sense: participatory urban sensing using a network of handheld air quality monitors. In: Proceedings of the 7th ACM Conference on Embedded Networked Sensor Systems, 2009. p. 349–350.

[20] Pousman Z, Stasko J, Mateas M (2007). Casual information visualization: Depictions of data in everyday life. IEEE Transact Visual Comput Graph.

[21] Chen M, Ebert D, Hagen H, Laramee RS, Van Liere R, Ma K-L, Ribarsky W, Scheuermann G, Silver D (2008). Data, information, and knowledge in visualization. IEEE Comput Graph Appl.

[22] Olshannikova E, Ometov A, Koucheryavy Y, Olsson T (2015). Visualizing big data with augmented and virtual reality: challenges and research agenda. J Big Data.

[23] Dourish P. Hci and environmental sustainability: the politics of design and the design of politics. In: Proceedings of the 8th ACM Conference on Designing Interactive Systems, 2010. p. 1–10.

[24] DiSalvo C, Sengers P, Brynjarsdóttir H. Mapping the landscape of sustainable hci. In: Proceedings of the SIGCHI Conference on Human Factors in Computing Systems, 2010. p. 1975–1984.

[25] Paulos E, Honicky R, Hooker B. Citizen science: Enabling participatory urbanism. In: Handbook of Research on Urban Informatics: The Practice and Promise of the Real-time City, 2009. p. 414–436. IGI Global.

[26] Rosi A, Mamei M, Zambonelli F, Dobson S, Stevenson G, Ye J. Social sensors and pervasive services: Approaches and perspectives. In: 2011 IEEE International Conference on Pervasive Computing and Communications Workshops (PERCOM Workshops), 2011. p. 525–530.

[27] Moloney J, Spehar B, Globa A, Wang R (2018). The affordance of virtual reality to enable the sensory representation of multi-dimensional data for immersive analytics: from experience to insight. J Big Data.

[28] Börner K, Record E. Macroscopes for making sense of science. In: Proceedings of the Practice and Experience in Advanced Research Computing 2017 on Sustainability, Success and Impact, 2017. p. 1–2.

[29] Saleem M, Valle HE, Brown S, Winters VI, Mahmood A (2018). The hiperwall tiled-display wall system for big-data research. J Big Data.

[30] Cecaj A, Lippi M, Mamei M, Zambonelli F (2021). Sensing and forecasting crowd distribution in smart cities: Potentials and approaches. IoT.

[31] Calabrese F, Pereira FC, Di Lorenzo G, Liu L, Ratti C. The geography of taste: analyzing cell-phone mobility and social events. In: International Conference on Pervasive Computing, 2010. p. 22–37. Springer

[32] Jiang S, Ferreira J, González MC (2017). Activity-based human mobility patterns inferred from mobile phone data: A case study of singapore. IEEE Transact Big Data.

[33] Mamei M, Bicocchi N, Lippi M, Mariani S, Zambonelli F (2019). Evaluating origin-destination matrices obtained from cdr data. Sensors.

[34] Wu Y, Wang L, Fan L, Yang M, Zhang Y, Feng Y (2020). Comparison of the spatiotemporal mobility patterns among typical subgroups of the actual population with mobile phone data: A case study of beijing. Cities.

[35] Balzotti C, Bragagnini A, Briani M, Cristiani E (2018). Understanding human mobility flows from aggregated mobile phone data. IFAC-PapersOnLine.

[36] Willberg E, Järv O, Väisänen T, Toivonen T (2021). Escaping from cities during the covid-19 crisis: Using mobile phone data to trace mobility in finland. ISPRS Int J Geo Information.

[37] Grantz KH, Meredith HR, Cummings DA, Metcalf CJE, Grenfell BT, Giles JR, Mehta S, Solomon S, Labrique A, Kishore N (2020). The use of mobile phone data to inform analysis of covid-19 pandemic epidemiology. Nature Commun.

[38] Santamaria C, Sermi F, Spyratos S, Iacus SM, Annunziato A, Tarchi D, Vespe M (2020). Measuring the impact of covid-19 confinement measures on human mobility using mobile positioning data. . a european regional analysis. Safety Sci.

[39] Traunmueller MW, Johnson N, Malik A, Kontokosta CE (2018). Digital footprints: Using wifi probe and locational data to analyze human mobility trajectories in cities. Comput Environ Urban Syst.

[40] Zhao F, Shi W, Gan Y, Peng Z, Luo X (2019). A localization and tracking scheme for target gangs based on big data of wi-fi locations. Cluster Comput.

[41] Soundararaj B, Cheshire J, Longley P (2020). Estimating real-time high-street footfall from wi-fi probe requests. Int J Geographical Informat Sci.

[42] Uras M, Cossu R, Ferrara E, Liotta A, Atzori L (2020). Pma: A real-world system for people mobility monitoring and analysis based on wi-fi probes. J Cleaner Prod.

[43] Potortì F, Crivello A, Girolami M, Barsocchi P, Traficante E (2018). Localising crowds through wi-fi probes. Ad Hoc Networks.

[44] Singh U, Determe J-F, Horlin F, De Doncker P (2020). Crowd forecasting based on wifi sensors and lstm neural networks. IEEE Transact Instrument Measur.

[45] Zhou Y, Lau BPL, Koh Z, Yuen C, Ng BKK (2020). Understanding crowd behaviors in a social event by passive wifi sensing and data mining. IEEE Internet Things J.

[46] Hong H, De Silva GD, Chan MC. Crowdprobe: Non-invasive crowd monitoring with wi-fi probe. Proceedings of the ACM on Interactive, Mobile, Wearable and Ubiquitous Technologies. 2018;2(3):1–23.

[47] Uras M, Cossu R, Ferrara E, Bagdasar O, Liotta A, Atzori L. Wifi probes sniffing: an artificial intelligence based approach for mac addresses de-randomization. In: 2020 IEEE 25th International Workshop on Computer Aided Modeling and Design of Communication Links and Networks (CAMAD), 2020. p. 1–6.

[48] Redondi AE, Cesana M (2018). Building up knowledge through passive wifi probes. Comput Commun.

[49] Cunche M, Kaafar M-A, Boreli R (2014). Linking wireless devices using information contained in wi-fi probe requests. Pervasive Mobile Comput.

[50] Andión J, Navarro JM, López G, Álvarez-Campana M, Dueñas JC (2018). Smart behavioral analytics over a low-cost iot wi-fi tracking real deployment. Wireless Commun Mobile Comput.

[51] Sagl G, Resch B, Hawelka B, Beinat E. From social sensor data to collective human behaviour patterns: Analysing and visualising spatio-temporal dynamics in urban environments. In: Proceedings of the GI-Forum, 2012;p. 54–63. Herbert Wichmann Verlag Berlin

[52] Silva TH, Viana AC, Benevenuto F, Villas L, Salles J, Loureiro A, Quercia D (2019). Urban computing leveraging location-based social network data: a survey. ACM Computing Surveys.

[53] da Mota VT, Pickering C (2020). Using social media to assess nature-based tourism: Current research and future trends. J Outdoor Recreation Tour.

[54] Silva TH, De Melo POV, Almeida JM, Loureiro AA (2014). Large-scale study of city dynamics and urban social behavior using participatory sensing. IEEE Wireless Commun.

[55] Wang D, Al-Rubaie A, Clarke SS, Davies J (2017). Real-time traffic event detection from social media. ACM Trans Internet Technol.

[56] Ghermandi A, Camacho-Valdez V, Trejo-Espinosa H (2020). Social media-based analysis of cultural ecosystem services and heritage tourism in a coastal region of mexico. Tour Manag.

[57] Devkota B, Miyazaki H, Witayangkurn A, Kim SM (2019). Using volunteered geographic information and nighttime light remote sensing data to identify tourism areas of interest. Sustainability.

[58] Preis T, Botta F, Moat HS (2020). Sensing global tourism numbers with millions of publicly shared online photographs. Environ Planning A Economy Space.

[59] Kádár B, Gede M (2021). Tourism flows in large-scale destination systems. Annals Tour Res.

[60] Ribeiro M, Nunes N, Nisi V, Schöning J (2020). Passive wi-fi monitoring in the wild: a long-term study across multiple location typologies. Personal Ubiquitous Comput.

[61] Wellmann T, Lausch A, Andersson E, Knapp S, Cortinovis C, Jache J, Scheuer S, Kremer P, Mascarenhas A, Kraemer R (2020). Remote sensing in urban planning: Contributions towards ecologically sound policies?. Landscape Urban Planning.

[62] Prandi C, Mirri S, Ferretti S, Salomoni P (2017). On the need of trustworthy sensing and crowdsourcing for urban accessibility in smart city. ACM Trans Internet Technol.

[63] Prandi C, Roccetti M, Salomoni P, Nisi V, Nunes NJ (2017). Fighting exclusion: a multimedia mobile app with zombies and maps as a medium for civic engagement and design. Multimedia Tools Appl.

[64] Longo A, Zappatore M, Bochicchio M, Navathe SB (2017). Crowd-sourced data collection for urban monitoring via mobile sensors. ACM Trans Internet Technol.

[65] Picaut J, Fortin N, Bocher E, Petit G, Aumond P, Guillaume G (2019). An open-science crowdsourcing approach for producing community noise maps using smartphones. Building Environ.

[66] Huang J, Duan N, Ji P, Ma C, Ding Y, Yu Y, Zhou Q, Sun W (2018). A crowdsource-based sensing system for monitoring fine-grained air quality in urban environments. IEEE Internet Things J.

[67] Golumbic YN, Fishbain B, Baram-Tsabari A (2019). User centered design of a citizen science air-quality monitoring project. Int J Sci Educ Part B.

[68] Loureiro P, Prandi C, Nunes N, Nisi V. Citizen science and game with a purpose to foster biodiversity awareness and bioacoustic data validation. In: Interactivity, Game Creation, Design, Learning, and Innovation, 2018;p. 245–255. Springer.

[69] Prandi C, Nisi V, Loureiro P, Nunes NJ (2020). Storytelling and remote-sensing playful interventions to foster biodiversity awareness. Int J Arts Technol.

[70] Niforatos E, Vourvopoulos A, Langheinrich M (2017). Understanding the potential of human-machine crowdsourcing for weather data. Int J Human Comput Stud.

[71] Njue N, Kroese JS, Gräf J, Jacobs S, Weeser B, Breuer L, Rufino M (2019). Citizen science in hydrological monitoring and ecosystem services management: State of the art and future prospects. Sci Total Environ.

[72] Sheppard SA, Turner J, Thebault-Spieker J, Zhu H, Terveen L. Never too old, cold or dry to watch the sky: A survival analysis of citizen science volunteerism. Proceedings of the ACM on Human-Computer Interaction 1(CSCW), 2017;1–21.

[73] Leonardi C, Cappellotto A, Caraviello M, Lepri B, Antonelli F. Secondnose: an air quality mobile crowdsensing system. In: Proceedings of the 8th Nordic Conference on Human-Computer Interaction: Fun, Fast, Foundational, 2014;p. 1051–1054.

[74] Tian R, Dierk C, Myers C, Paulos E. Mypart: Personal, portable, accurate, airborne particle counting. In: Proceedings of the 2016 CHI Conference on Human Factors in Computing Systems, 2016;p. 1338–1348.

[75] Kobernus MJ, Berre A-J, Gonzalez M, Liu H-Y, Fredriksen M, Rombouts R, Bartonova A. A practical approach to an integrated citizens’ observatory: The citi-sense framework. 2015.

[76] Luna S, Gold M, Albert A, Ceccaroni L, Claramunt B, Danylo O, Haklay M, Kottmann R, Kyba C, Piera J. *et al*. Developing mobile applications for environmental and biodiversity citizen science: considerations and recommendations. In: Multimedia Tools and Applications for Environmental & Biodiversity Informatics, 2018;p. 9–30. Springer.

[77] Pejovic V, Skarlatidou A (2020). Understanding interaction design challenges in mobile extreme citizen science. Int J Human Comput Interaction.

[78] Pataki BA, Garriga J, Eritja R, Palmer JR, Bartumeus F, Csabai I (2021). Deep learning identification for citizen science surveillance of tiger mosquitoes. Scientific Rep.

[79] Brown C, Chauhan J, Grammenos A, Han J, Hasthanasombat A, Spathis D, Xia T, Cicuta P, Mascolo C. Exploring automatic diagnosis of covid-19 from crowdsourced respiratory sound data. In: Proceedings of the 26th ACM SIGKDD International Conference on Knowledge Discovery & Data Mining, 2020;p. 3474–3484.

[80] Wang P, Lin C, Obaidat MS, Yu Z, Wei Z, Zhang Q. Contact tracing incentive for covid-19 and other pandemic diseases from a crowdsourcing perspective. IEEE Internet of Things Journal. 2021. 10.1109/JIOT.2020.3049024 PMC8768981 35782186

[81] Valkanova N, Jorda S, Moere AV (2015). Public visualization displays of citizen data: design, impact and implications. Int J Human Comput Stud.

[82] Moere AV, Hill D (2012). Designing for the situated and public visualization of urban data. J Urban Technol.

[83] Valkanova N, Jorda S, Tomitsch M, Vande Moere A. Reveal-it! the impact of a social visualization projection on public awareness and discourse. In: Proceedings of the SIGCHI Conference on Human Factors in Computing Systems, 2013;p. 3461–3470.

[84] Claes S, Coenen J, Vande Moere A. Empowering citizens with spatially distributed public visualization displays. In: Proceedings of the 2017 ACM Conference Companion Publication on Designing Interactive Systems, 2017;p. 213–217.

[85] Hsu Y-C, Dille P, Cross J, Dias B, Sargent R, Nourbakhsh I. Community-empowered air quality monitoring system. In: Proceedings of the 2017 CHI Conference on Human Factors in Computing Systems, 2017;p. 1607–1619.

[86] Prandi C, Ceccarini C, Nisi V, Salomoni P. Designing interactive infographics to stimulate environmental awareness: an exploration with a university community. Multimedia Tools and Applications. 2020;1–18.

[87] Ramachandran GS, Bogosian B, Vasudeva K, Sriramaraju SI, Patel J, Amidwar S, Malladi L, Shylaja RD, Kumar NRB, Krishnamachari B. An immersive visualization of micro-climatic data using usc air. In: Proceedings of the 17th Annual International Conference on Mobile Systems, Applications, and Services, 2019;p. 675–676.

[88] Eldin DM, Hassanien AE, Hassanien EE. Challenges of big data visualization in internet-of-things environments. In: International Conference on Innovative Computing and Communications, 2020;p. 873–885. Springer

[89] Protopsaltis A, Sarigiannidis P, Margounakis D, Lytos A. Data visualization in internet of things: tools, methodologies, and challenges. In: Proceedings of the 15th International Conference on Availability, Reliability and Security, 2020;p. 1–11.

[90] Lavalle A, Teruel MA, Maté A, Trujillo J (2020). Improving sustainability of smart cities through visualization techniques for big data from iot devices. Sustainability.

[91] Cairns P. Doing Better Statistics in Human-computer Interaction. Cambridge University Press, 2019.

[92] Grace K, Wasinger R, Ackad C, Collins A, Dawson O, Gluga R, Kay J, Tomitsch M. Conveying interactivity at an interactive public information display. In: Proceedings of the 2nd ACM International Symposium on Pervasive Displays, 2013;p. 19–24.

[93] Peltonen P, Kurvinen E, Salovaara A, Jacucci G, Ilmonen T, Evans J, Oulasvirta A, Saarikko P. It’s mine, don’t touch! interactions at a large multi-touch display in a city centre. In: Proceedings of the SIGCHI Conference on Human Factors in Computing Systems, 2008;p. 1285–1294.

[94] Müller J, Wilmsmann D, Exeler J, Buzeck M, Schmidt A, Jay T, Krüger A. Display blindness: The effect of expectations on attention towards digital signage. In: International Conference on Pervasive Computing, 2009;p. 1–8. Springer

[95] Parra G, Klerkx J, Duval E. Understanding engagement with interactive public displays: an awareness campaign in the wild. In: Proceedings of The International Symposium on Pervasive Displays, 2014;p. 180–185.

[96] Coenen J, Claes S, Moere AV. The concurrent use of touch and mid-air gestures or floor mat interaction on a public display. In: Proceedings of the 6th ACM International Symposium on Pervasive Displays, 2017;p. 1–9.

